# Development of Yorkshire Terrier Dentition

**DOI:** 10.3390/vetsci10070406

**Published:** 2023-06-21

**Authors:** Corrin Wallis, Francesca Solmi, Ilaria Pesci, Neil Desforges, Lucy J. Holcombe

**Affiliations:** 1 Waltham Petcare Science Institute, Waltham-on-the-Wolds, Leicestershire LE14 4RT, UK; 2 Interuniversity Institute for Biostatistics and Statistical Bioinformatics (I-Biostat), Data Science, Hasselt University, 3500 Hasselt, Belgium

**Keywords:** dentition development, dog, Yorkshire terrier, persistent deciduous teeth, body weight

## Abstract

**Simple Summary:**

Several problems, particularly in smaller breed dogs, have been associated with tooth development, e.g., over-crowding, missing teeth, and persistent deciduous teeth (where a juvenile tooth remains in the mouth at the same time as its permanent counterpart). This opportunistic study of 61 Yorkshire terrier puppies aimed to determine the average age of deciduous tooth loss and permanent tooth eruption, the incidence of persistent deciduous teeth, and potential influencing factors such as body weight. The age at which deciduous teeth were lost, and permanent teeth erupted, varied according to body weight; dogs weighing less than 3 kg lost their deciduous teeth and gained their permanent teeth later than dogs weighing more than 3 kg. The sequence of tooth loss and eruption (i.e., incisors first followed by canines, premolars, and then molars) was disrupted in dogs < 3 kg. Persistent deciduous teeth were common, affecting 69% of puppies. Those with lower body weights (<3 kg) had greater proportions and were more likely to require surgical extractions by a veterinarian. This study highlights the importance of regular checks by a veterinary professional during the active phases of tooth development.

**Abstract:**

The development of dentition in dogs has been associated with several problems including tooth over-crowding, missing permanent dentition, and persistent deciduous teeth (PDT). Information on dentition development in different breeds is lacking. This study of 61 Yorkshire terriers aimed to determine the (i) average age at deciduous tooth exfoliation, (ii) average age at permanent tooth eruption, (iii) PDT incidence, and influencing factors such as body weight. The ages of exfoliation of deciduous teeth and eruption of permanent dentition were influenced by body weight and tooth type. These dentition changes tended to occur later in dogs ≤ 3 kg versus dogs > 5 kg. Generally, incisors were exfoliated first, followed by premolars and then canines. At a body weight of 4.5 kg, the middle of the data range, the estimated age at loss of deciduous teeth (with 95% confidence intervals) was 21.9 (21.1, 22.9) weeks for incisors, 26.1 (24.9, 27.4) weeks for canines, and 23.9 (22.9, 24.9) weeks for premolar. The estimated age at eruption of permanent dentition was 22.3 (21.6, 23.0) weeks for incisors, 23.8 (23.0, 24.6) weeks for canines, 24.7 (24.0, 25.5) weeks for premolars, and 26.4 (25.5, 27.3) for molar teeth. However, this sequence was disrupted in dogs ≤ 3 kg. Yorkshire terriers had a high incidence of PDT. At a body weight of 4.5 kg, the estimated proportion of PDT was: incisors 0.86% (0.32, 2.31), canines 15.62% (7.62, 29.37) and premolars 3.57% (1.62, 7.66). Canines constituted the most frequently retained tooth type, with 89.1% retained in dogs ≤ 3 kg compared to 12.0% in dogs > 5 kg. This information will enable veterinarians to provide personalised advice regarding the oral care requirements for Yorkshire terriers and highlights the need to regularly monitor this breed between the ages of two and seven months, during the active phases of tooth development.

## 1. Introduction

Dogs have two sets of teeth over the course of their lifetimes (i.e., they are diphyodonts). The deciduous teeth begin to erupt at three to six weeks of age, and by twelve weeks of age, most breeds of dog will have their full complement of 28 teeth [1,2,3]. As the permanent teeth develop, they place pressure on the apex of the deciduous tooth, causing the resorption of the deciduous tooth root. This process continues until the deciduous root is sufficiently resorbed, the tooth is exfoliated, and the permanent tooth occupies its position. The permanent dentition begins to erupt at about three months of age and most breeds of dog will have their full complement of 42 permanent teeth by seven months of age [1,2,3]. A number of factors are thought to affect tooth eruption including genetics, infections, and trauma [1,2,3].

Several problems have been associated with the development of dentition, such as delayed eruption, over-crowding, missing underlying permanent dentition, and PDT [3]. A deciduous tooth is termed persistent if it exists in the mouth at the same time as its permanent counterpart [4]. PDT is particularly common in small and toy breeds of dog [4,5,6]. A prevalence rate of 5.7% was reported in a UK study of 11,647 Chihuahuas, increasing to 11.3% in individuals less than two years of age [7]. The most frequent cause of PDT is an incorrect eruption path of the permanent tooth [4]. PDT and the subsequent tooth over-crowding create regions in the mouth where plaque can accumulate, which can lead to the development of periodontal disease [4,8]. They can also cause orthodontic issues; for example, the mandibular canines can impinge on the palate [4,8,9]. These problems have been reported to occur soon after the permanent canines start to erupt [8]. Abnormal tooth eruption can also cause malocclusion (misalignment of teeth during bite), which can be mild to severe and may require correction [9]. Treatment options include the extraction of the offending tooth, removal of the crown combined with endodontic therapy, and orthodontics to move the teeth into the correct position. These tooth eruption anomalies are thought to be genetic due to the pattern of occurrence within different breeds of dog.

There is a lack of published information on variation in the age and sequence of permanent tooth eruption, and incidence of PDT, between different breeds of dog. An opportunity arose to study a population of Yorkshire terriers, a susceptible breed to dental issues, that were being acquired by the Waltham Petcare Science Institute. This breed was selected as a representative breed of the toy dog category based on its popularity, medical predispositions, behaviour, and genetic diversity. The primary objectives of the study were to determine (i) the average age when deciduous teeth are exfoliated, (ii) the average age when permanent teeth erupt, (iii) and the incidence of PDT in Yorkshire terriers. A secondary objective was to investigate the influence of factors such as body weight and litter. This information will enable veterinarians to provide personalised advice regarding the oral care requirements for Yorkshire terriers.

## 2. Materials and Methods

### 2.1. Study Cohort

A total of 61 Yorkshire terriers (14 litters) were acquired, between September 2012 and April 2016, from registered external breeders by the Waltham Petcare Science Institute. There were no inclusion or exclusion criteria for the selection of breeders other than being based in the United Kingdom and their willingness to supply pure-bred Yorkshire terrier puppies. All puppies included in the study had genetic DNA tests (Wisdom Panel™, Mars Petcare, Portland, OR, USA) conducted on them which confirmed that they were representative of the global Yorkshire terrier pet population. The pure-bred Yorkshire terrier puppies were from 11 bitches, each of which had mated with one of eight dogs. The puppies were housed in pairs in environmentally enriched kennels and provided with a comprehensive socialisation programme. Thirty of the dogs were female and 31 were male. The female dogs were intact and the male dogs were castrated between 24.4 and 29.4 weeks of age. The average weight of the dogs at 28 weeks of age was 4.54 kg (range 1.53 to 7.55 kg). There were 21 dogs ≤ 3.0 kg, 24 dogs between >3.0 kg and ≤5.0 kg, and 16 dogs > 5.0 kg. Body weight was not recorded for five dogs (one litter) at 28 weeks of age as they had been re-homed. The dogs were fed a commercial dry diet (Royal Canin^®^ Yorkshire terrier 29 Junior) from weaning up to 14 weeks of age. From 14 to 28 weeks of age, 32 dogs remained on the dry diet, 8 were transitioned onto a commercial wet diet (Cesar^®^ puppy with chicken and rice with a carrot topping), and 21 were fed a simultaneous offering of the two.

### 2.2. Study Design

The status of each dog’s primary and permanent dentition was assessed and recorded at 12, 16, 20, 24, and 28 weeks of age (±2 weeks). The various tooth states included erupted (E), not erupted (N), partially erupted (P), not visible (X), and lost/exfoliated (L). The information was captured using a modified dental chart and later entered into a bespoke Microsoft^®^ Access^®^ database. Dogs received regular mouth handling from 8 to 10 weeks of age so that their dentition could be visualised without the need for anaesthesia. Six people performed assessments, and all were trained by recognised European Specialists in Veterinary Dentistry (Peter Southerden, Eastcott Dental Referrals, Swindon, Wiltshire, UK and Lisa Milella, The Veterinary Dental Surgery, Byfleet, Surrey, UK). Any PDT identified were surgically extracted at 32 weeks of age, or sooner if deemed necessary by the European Specialists in Veterinary Dentistry operating under the Veterinary Surgeons Act.

### 2.3. Data Handling

The variables recorded as part of the study are summarised in Table 1. Since oral assessments were performed at four-weekly intervals, and so the exact date of loss/eruption was unknown, the data is effectively “interval censored”. For the purposes of these analyses, the average at the censoring interval was used to estimate age at exfoliation/eruption.

The deciduous teeth data was utilised to determine the age at loss of deciduous dentition. The average age, in weeks, between the last time point at which the tooth status was ‘L’ and the first time point at which the tooth status was ‘E’ or ‘P’ was determined. If the tooth had not been exfoliated at the last age recorded, it was labelled as censored. The data were assigned as missing if the status was always ‘X’, ‘X’ occurred before ‘L’, ‘X’ was last measured after ‘E’ or ‘P’, or ‘E’ or ‘P’ were recorded after ‘L’.

The permanent teeth sub-data set was utilised to determine the age at the eruption of permanent dentition. The average age, in weeks, between the last time point at which the tooth status was ‘N’ and the first time point at which the tooth status was ‘E’ or ‘P’ was determined. The last age recorded was assigned if the tooth status was never ‘E’, and the value was labelled as censored. The data was assigned as missing if the status was always ‘X’, if ‘X’ occurred before ‘E’ or ‘P’, if ‘X’ was the last measure after ‘N’, or if there were later measurements labelled as ‘N’ after an ‘E’ or ‘P’.

### 2.4. Statistical Modelling

Data were analysed using the statistical software R 3.5 using lme4, multcomp, and survival packages [10,11,12,13].

Survival regression models were used for both age at loss of deciduous dentition and age at eruption of permanent dentition. The age at loss/eruption was used as the response variable, each dog was used as a frailty term, and body weight, tooth type (or tooth number), and their interaction were considered fixed effects. The effect of body weight was added as a linear and quadratic effect. The interaction of body weight and tooth type was initially added on both linear and quadratic body-weight terms, but the model was then simplified if required. Both tooth type models contained the main effects of tooth type and body weight in both linear and quadratic terms, and all interactions were dropped from the permanent eruption but not the deciduous loss tooth type model. The tooth number deciduous loss model contained the main effects of tooth number and body weight, in linear and quadratic terms, and the interaction between the tooth number and the linear effect of weight. The tooth number permanent eruption model contained both the main effects of tooth number and body weight in linear and quadratic terms as well as all interactions. The models used assumed Weibull distributions for the log(age) values and normal distributions for the frailty terms. Multiplicity correction was carried out using a single-step correction method based on the joint normal distribution of the linear function of the model parameters [11].

Using mixed logistic regression models, the events of PDT and surgical extraction were used as response variables. Litter and dog nested in litter were included as the random effects. Body weight, tooth type, and their interaction were included as fixed effects. For both analyses, the random litter effect was dropped from the model as it was not significant (*p* > 0.05). The effect of body weight was added as a linear and quadratic effect. The interaction of body weight and tooth type was initially added on both linear and quadratic body weight terms, but the model was then simplified and only the interaction term with the linear body weight effect was retained. The final models contained a significant interaction effect of body weight and tooth type (*p* < 0.05).

## 3. Results

The development of dentition in Yorkshire terriers from 14 litters was investigated. The age at exfoliation of 1708 deciduous teeth (28 teeth, 61 dogs) and the age at eruption of 2562 permanent teeth (42 teeth, 61 dogs) were recorded based on assessments every 4 weeks from 12 to 28 weeks of age. In addition, the incidence of PDT was determined and, if surgical extraction was necessary, a record was made, and these data were included in the analysis. There were missing data points for four dogs due to difficulties assessing their teeth for behavioural reasons: two dogs had data missing for one time point, one dog had two missing time points, and one dog had three missing time points. Five dogs were removed from the trial at 28 weeks of age, due to being re-homed, and so were omitted from the persistent teeth analysis.

### 3.1. Age at Exfoliation of Deciduous Dentition

**Figure 1:**
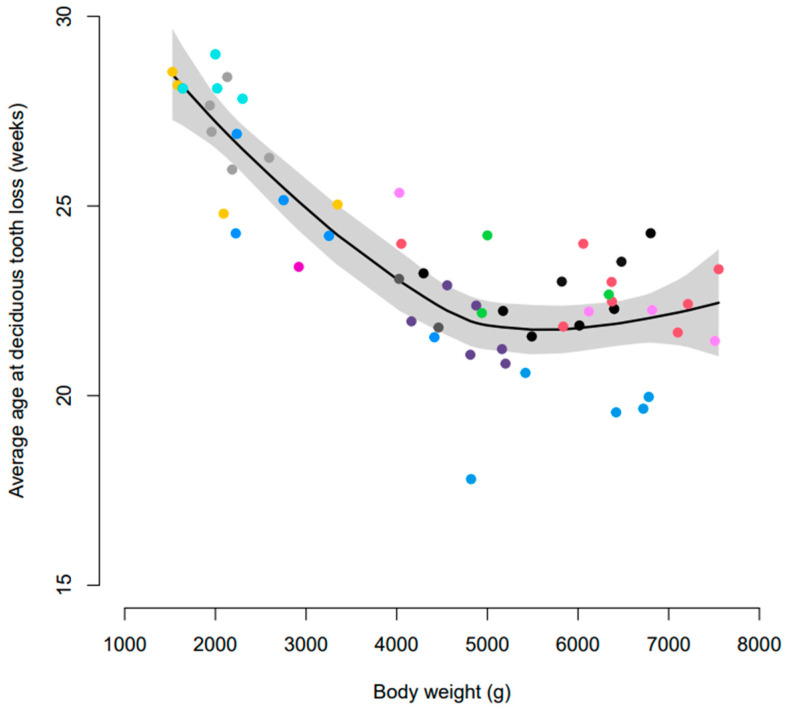
Smoothed average age representation of deciduous tooth loss (mouth average) according to body weight at 28 weeks of age. Coloured dots represent individual litters and the shaded area represents 95% confidence intervals.

The initial inspection of the data suggested that the age at exfoliation of deciduous dentition varied according to litter; however, this was confounded by body weight. There was also a relationship between the average age of exfoliation of deciduous teeth and body weight (Figure 1). Preliminary exploration of the data using arbitrary body weight groups showed that, on average, dogs ≤ 3 kg exfoliated their primary dentition later than dogs > 3 kg (Table 2). Various tooth types were also exfoliated at different ages. Generally, the incisors were exfoliated first, followed by the premolars and canines (Table 2).

**Figure 2:**
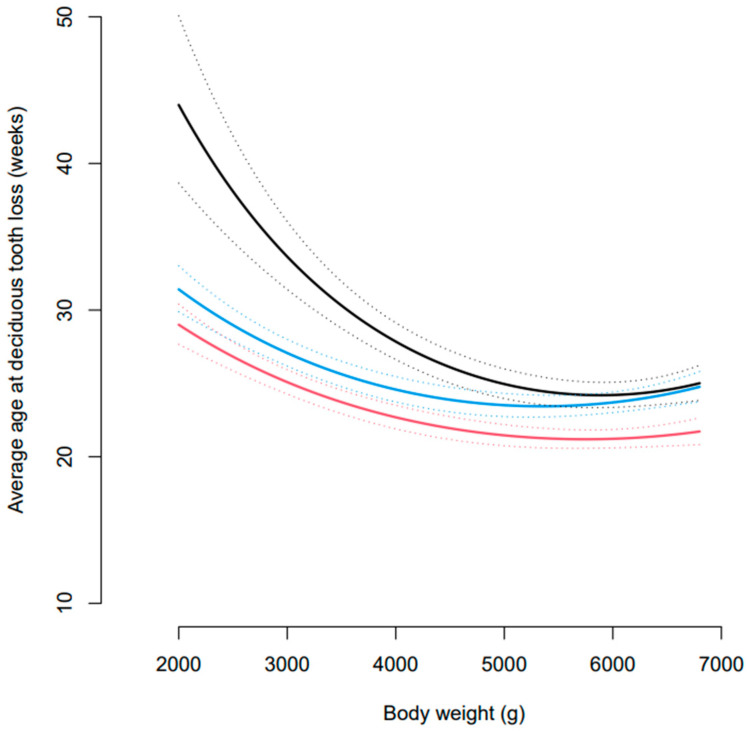
Estimated average age of deciduous tooth loss for incisors (red), canines (black), and premolar (blue) teeth across a range of body weights seen in the cohort. Dotted lines represent pointwise 95% confidence intervals.

Statistical modelling of the data showed that the effect of body weight on the average age at exfoliation of deciduous dentition differed based on tooth type (Figure 2). At body weights of 2 kg and 4.5 kg, all tooth types significantly differed in terms of the age at which they were exfoliated (all *p* < 0.0001). However, at 7 kg the incisors significantly differed to the canines and premolars (both *p* < 0.00001), but the premolars and canines did not significantly differ (*p* = 0.87). According to the statistical model, at a body weight of 4.5 kg, the middle of the data range, the estimated age at loss of deciduous teeth (with 95% confidence intervals) was 21.9 (21.1, 22.9) weeks for incisors, 26.1 (24.9, 27.4) weeks for canines, and 23.9 (22.9, 24.9) weeks for premolars.

The estimates for the mean age at deciduous tooth loss for each tooth in a dog weighing 4.5 kg showed that the mandibular first (701, 801) and second incisors (702, 802) were the first teeth to be exfoliated (Supplementary Table S1). The mandibular third incisors (703, 803) and then the maxillary first (501, 601) and second incisors (502, 602) closely followed this. The mandibular and maxillary third and fourth premolars (507,508, 607, 608, 707, 708, 807, 808) were the next teeth to be exfoliated, followed by the maxillary third incisors (503, 603). The mandibular and maxillary second premolars (506, 606, 706, 806) and mandibular canines (704, 804) were exfoliated next, and the final teeth to be exfoliated were the maxillary canines (504, 604).

### 3.2. Age at Eruption of Permanent Dentition

**Figure 3:**
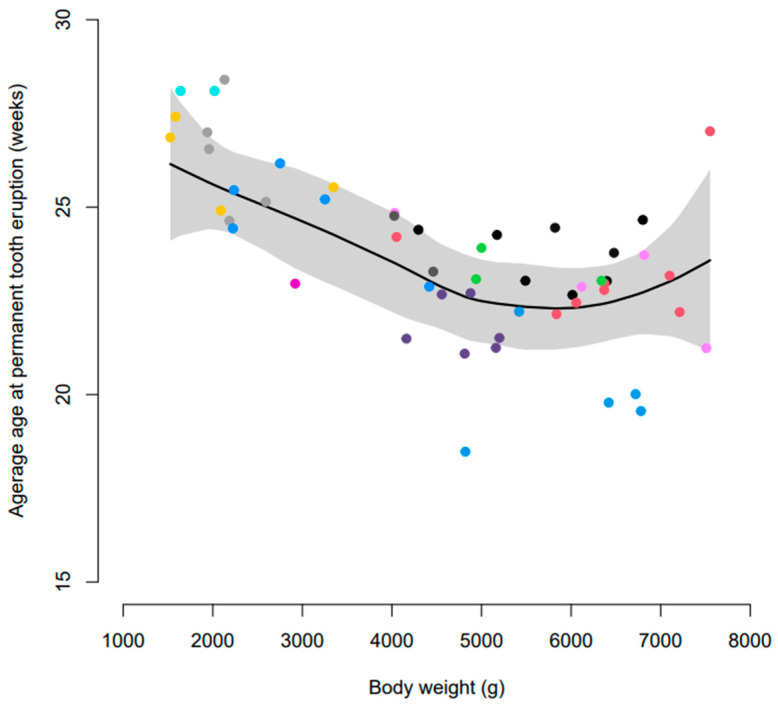
Smoothed average age at eruption of permanent dentition according to body weight. Coloured dots represent individual litters and the shaded area represents built-in 95% confidence intervals.

**Figure 4:**
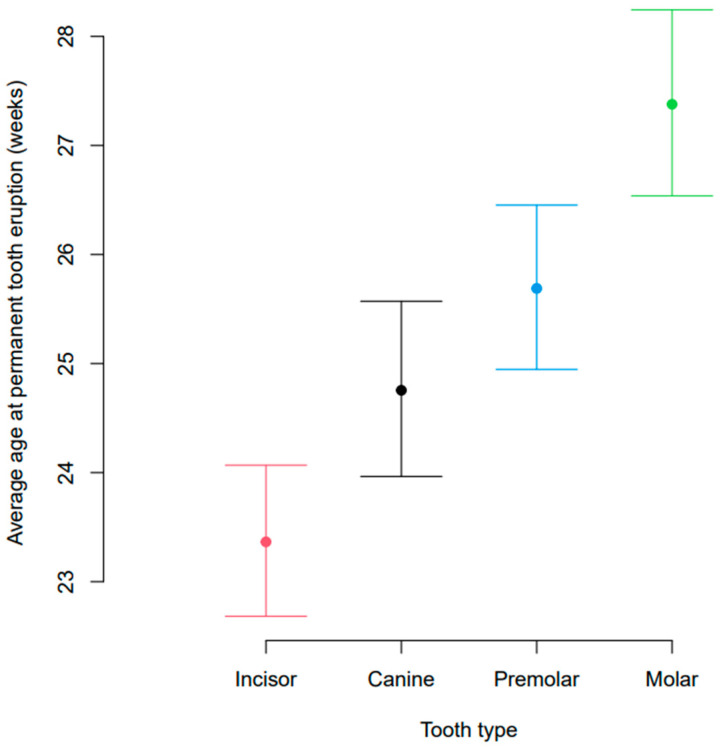
Average age at eruption of permanent dentition for each of the different tooth types (incisor, canine, premolar, and molar). The bars indicate 95% confidence intervals.

Initial assessment of the data suggested that the age at eruption of permanent dentition was subject to dog-to-dog variation. There was also a relationship between body weight and age at eruption of permanent dentition (Figure 3). Generally, the eruption of permanent dentition was later in dogs ≤ 3 kg compared to those >3 kg (Table 3). Overall, incisors erupted first, followed by canines, premolars, and molars in dogs > 3 kg (Figure 4). However, in dogs ≤ 3 kg, the eruption sequence changed to incisors, premolars, molars, and then canines (Table 3).

**Figure 5:**
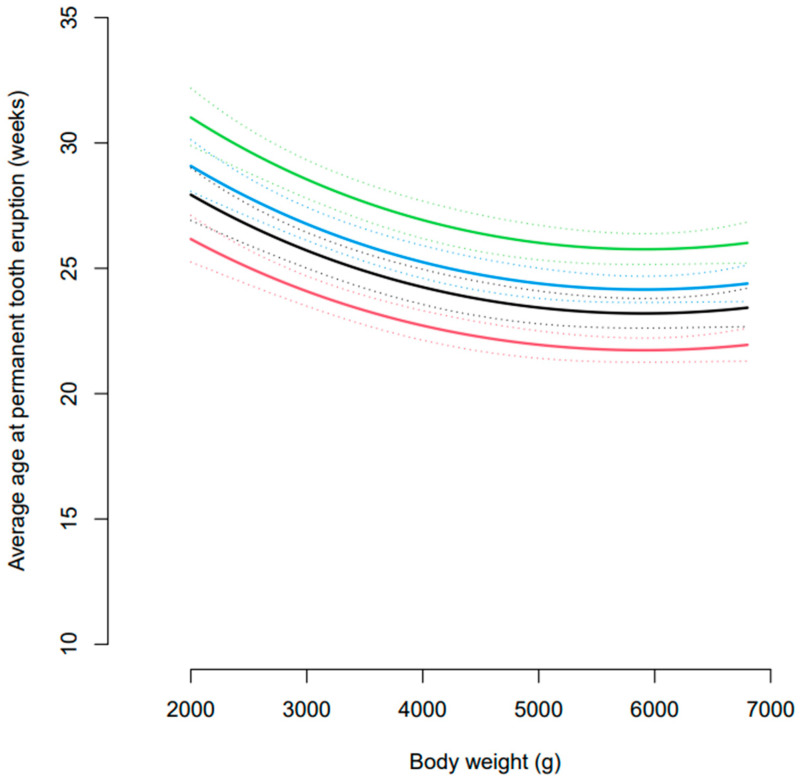
Estimated average age at eruption of permanent dentition for incisors (red), canines (black), premolars (blue), and molars (green) teeth across a range of body weights. The dotted lines represent 95% confidence intervals.

Statistical modelling of the data showed a significant effect of body weight and of tooth type on the estimated age at tooth eruption (Figure 5). At a body weight of 4.5 kg, the middle of the data range, the estimated age at eruption of permanent dentition was 22.3 (21.6, 23.0) weeks for incisors, 23.8 (23.0, 24.6) weeks for canines, 24.7 (24.0, 25.5) weeks for premolars, and 26.4 (25.5, 27.3) for molar teeth. The age at tooth eruption significantly differed between all four tooth types (all *p* < 0.0001) and these differences were constant across the different body weights. There was no significant interaction between body weight and tooth type in the final statistical model, and therefore, the comparison by tooth type was the same for all body weights.

On the basis of statistical model estimates, the first permanent teeth to erupt, at a body weight of 4.5 kg, were the maxillary and mandibular first incisors (101, 201, 301, 401) (Supplementary Table S2). The maxillary and mandibular second incisors (102, 202, 302, 402), mandibular third incisors (303, 403), and the maxillary fourth premolars (108, 208) followed this. Next to erupt were the mandibular first molars (309, 409), maxillary third premolars (107, 207), maxillary and mandibular canines (104, 204, 304, 404), and mandibular fourth premolars (308, 408). These were followed by the mandibular third premolars (307, 407), maxillary first premolars (105, 205) and first molars (109, 209), and maxillary third incisors (103, 203). Subsequently, the mandibular and maxillary second premolars (106, 206, 306, 406), second molars (110, 210, 310, 410), and mandibular first premolars (305, 405) erupted. The final teeth to erupt were the mandibular third molars (311, 411).

### 3.3. Incidence of PDT

**Figure 6:**
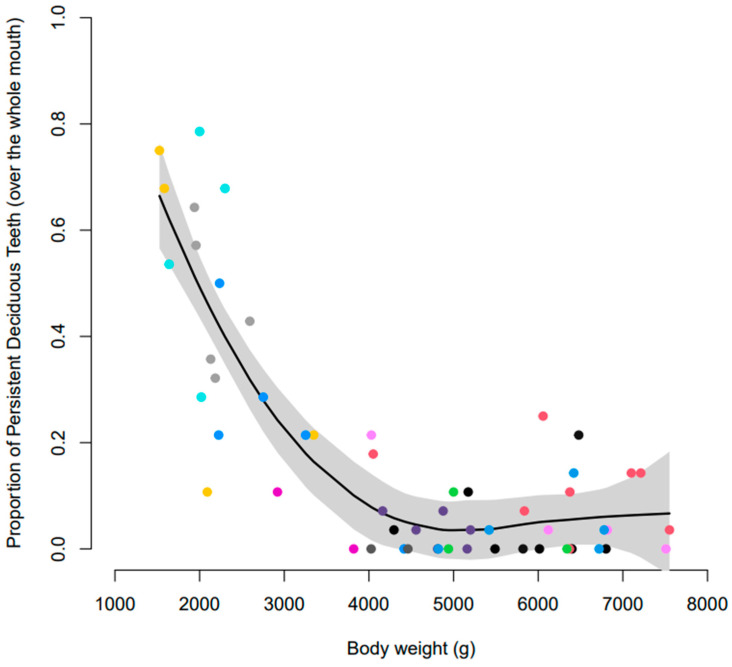
Smoothed mean proportion of PDT according to body weight. Coloured dots represent individual litters and the shaded area represents 95% confidence intervals.

Preliminary assessment of the data indicated that 42 of the 61 Yorkshire terriers (69%) had one or more PDT. There was dog-to-dog variability in the number of PDT, varying from 1 to 22. There was also an effect of body weight wherein dogs with lower body weights (≤3 kg) had a greater proportion of PDT than those with higher body weights (>3 kg) (Figure 6; Table 4). The proportion of PDT that were not naturally exfoliated, and required surgical extraction, was also greater for dogs with lower body weights than heavier dogs (Table 5). There were also differences between tooth types; canine teeth were most likely to be retained, followed by premolar and then incisor teeth (Table 4 and Table 5). There were 22 dogs that were recorded as having overshot jaws (class 2 malocclusion) and 10 with undershot jaws (class 3 malocclusion) at one or more points throughout the study. The proportion of dogs requiring surgical extractions was similar between those with malocclusions recorded (62.5%) versus those with normal occlusions (58.6%).

**Figure 7:**
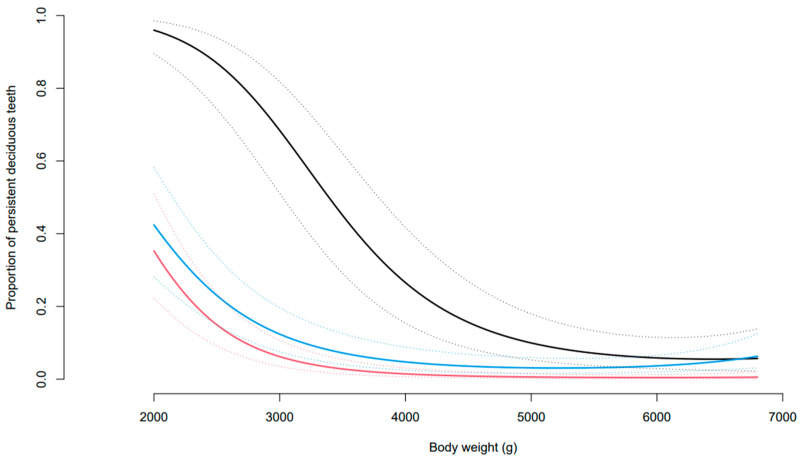
Estimated mean proportion of PDT by body weight and tooth type: incisor (red), canine (black), and premolar (blue). Dotted lines represent 95% confidence intervals.

Statistical modelling showed that the effect of body weight on the proportion of PDT differed significantly by tooth type (Figure 7). At a body weight of 4.5 kg, the middle of the data range, the estimated proportion of PDT in each tooth type was: incisors 0.86% (0.32, 2.31), canines 15.62% (7.62, 29.37), and premolars 3.57% (1.62, 7.66). The odds of a canine being retained were 21.3 (8.9, 51.1) times that of an incisor and 5.0 (2.7, 9.4) times that of a premolar (both *p* < 0.00001) being retained. The odds of a persistent premolar being retained were 4.3 (2.0, 9.1) times that of an incisor (*p* = 0.0002). When only those that required surgical extraction are considered, the estimated proportions were 0.24%, (0.05, 1.22) for incisors, 6.63% (2.19, 18.36) for canines, and 2.23% (0.72, 6.73) for premolars.

Dogs with lower body weights (2.0 kg) had an increased estimated proportion of PDT for all types of teeth: incisors 35.29% (20.19, 54.05), canines 95.97% (87.34, 98.91), and premolars 42.42% (25.67, 61.15). When considering heavier dogs (7 kg), the estimated proportion of PDT decreased for all tooth types: incisors 0.61% (0.11, 3.25), canines 5.99% (1.72, 18.87), and premolars 7.6% (2.91, 18.46). At a body weight of 2 kg, the canine teeth were significantly more likely to be retained than the incisors and premolars (both *p* < 0.00001), but the incisor and premolar teeth did not significantly differ (*p* = 0.39). At a body weight of 7 kg, the canines and premolars were significantly more likely to be retained than the incisor teeth (*p* = 0.0035 and *p* < 0.00001, respectively), but the premolars and canines did not significantly differ (*p* = 0.85).

## 4. Discussion

To our knowledge, this is the first study to report on the age at exfoliation, age at eruption, and prevalence of PDT in Yorkshire terriers. Our findings suggest that several different factors could affect tooth development such as litter and body weight.

Visual inspection of the data indicated litter-to-litter variability in the proportion of PDT. Although litter was confounded by body weight, a role for genetics in the development of dentition in this breed of dog cannot be ruled out. No published studies were identified which had investigated the role of genetics in tooth development in dogs. However, studies of human twins have suggested a strong genetic basis for the development of dentition [14,15,16,17]. A genome-wide association study of the number of permanent teeth erupted between ages six and fourteen, using records from women in the Danish National Birth cohort, identified loci that were strongly associated with tooth eruption [18]. A population-based genome-wide association study of individuals from the Avon Longitudinal Study of Parents and Children and the 1966 Northern Finland Birth Cohort identified loci associated with time to first tooth eruption and number of teeth at one year of age [19,20]. Some publications suggest that heritability is higher for tooth development than for tooth eruption [21,22]. These studies have not determined whether the developments of primary and permanent dentition have different or similar mechanisms. Furthermore, the mutations identified may simply be markers of the stage of overall maturation rather than being specifically associated with dental development. Further work is required to determine the role of genetics in the development of canine dentition. Longitudinal and cross-sectional studies of humans have also reported differences in tooth emergence time among different races [23,24,25,26,27,28,29,30,31,32,33,34,35]. There is a lack of information regarding differences between breeds of dog in their age at tooth emergence and loss. Again, further research is required to determine whether there is a breed effect on the age and sequence of tooth eruption.

Body weight could explain the patterns observed across the litters in the age at exfoliation of deciduous teeth, age at eruption of permanent dentition, and prevalence of PDT. This study has shown that the age of deciduous tooth loss and permanent tooth eruption in dogs negatively correlates with body weight. It would be interesting to understand whether smaller size and body weight are consequences of tooth development issues (e.g., affecting food intake) or whether these issues arise due to genetic predispositions to smaller size consequently impacting dental development (e.g., tooth over-crowding). These findings show some similarity with human studies that have shown a negative linear correlation between the time of first deciduous tooth eruption and birth weight [36,37]. Delayed tooth eruption has also been reported in low-body-weight infants and premature infants [38,39]. Infant age and weight have also been shown to independently influence the timing of tooth eruption [40,41,42]. In contrast, other studies were non-conclusive about the relationship between body weight, or birth height, and age at first dental eruption [43,44,45]. Dogs with a body weight ≤ 3 kg also had a greater proportion of PDT than those >3 kg. The different sequence of permanent tooth eruption in dogs ≤ 3 kg when compared with larger dogs is likely to be directly related to the higher incidence of PDT in Yorkshire terriers.

Tooth over-crowding may also play a role in the development of dentition in dogs. Human studies have shown that tooth eruption is earlier when there is adequate space within the jaw bone [28]. This theory may also hold true for dogs, as toy and small breed dogs have been reported to have more crowded adult dentition than larger breeds [46,47,48]. A contributing factor in this may be the reported proportionately larger mandible first molar relative to mandibular height in small versus large dogs [49]. Toy/small- and medium/large-breed dogs also significantly differ in the oral biometrics of their upper mandibles (Pesci et al., personal communication). The total mesio-distal length:tooth arch ratio has been shown to be higher in smaller breeds, indicating that there is less space between teeth. This finding concurs with a study of jaws and teeth from a collection of 250 dogs [50]. Toy/small-breed dogs also have significantly larger teeth relative to their arch perimeter compared with medium/large dogs (Pesci et al., personal communication). It is possible that the selective breeding of smaller dogs has selected for mutations in genetic loci that regulate body size. One hypothesis is that this disconnect between tooth and jaw size may suggest that the genetic factors that influence body size are independent from those that regulate the size of dentition. A study of monozygotic and dizygotic twins indicated a strong genetic influence on tooth dimensions in humans [51].

In total, 69% of the Yorkshire terriers had one or more PDT and 76% of these required surgical extractions under general anaesthesia based on the recommendation of European Specialists in Veterinary Dentistry. This was higher than observed in other studies with the exception of what has been reported for toy poodles. A retrospective analysis of medical records of 759 dogs aged 7–18 months reported an overall incidence of 19.7% [52]. In a study of 259 dogs (representing 23 breeds), based on a retrospective analysis of radiographs, the overall prevalence was 5.4% with all cases falling within the 7-month to 2-year age category [53]. The latter study reported breed differences in the prevalence of PDT; 74.4% of the cases observed were toy poodles, 11.2% Chihuahuas, and 10% were other small dogs. A study of health problems in 11,647 Chihuahuas aged 0.1–18.6 years showed an overall PDT prevalence of 5.7%, rising to 11.3% in dogs aged less than two years [7]. An examination of 251 mongrels, including 143 strays kept in animal protection offices in Tokyo, identified 46 dogs (18.3%) less than 0.5 years old with PDT [54]. In this study of Yorkshire terriers, canines were the most likely teeth to be retained. Others have also reported canines as the most frequently affected teeth in dogs. At an average body weight of 4.5 kg, 15.6% of canines were retained, and this number rose to 96.0% for dogs < 2 kg [53]. Human studies indicate that the most common types of persistent primary teeth are the mandibular second molars and maxillary canines [55,56]. PDT can cause the permanent teeth to erupt in abnormal positions, which can result in an abnormal jaw position, the over-crowding of teeth, accidental bites into the palate, and an increased predisposition to periodontitis. To prevent complications, once the permanent tooth has erupted, its deciduous counterpart should be surgically extracted.

Several human studies reported that the mandibular permanent teeth erupted earlier than maxillary opposing teeth, sometimes with the exception of the premolars and molars [29,57,58,59]. In Yorkshire terriers, there was no clear trend, with teeth simultaneously erupting on the maxillae and mandibles. For all body weights, the first permanent teeth to erupt were the mandibular and maxillary incisors, followed by the maxillary fourth premolars. Amongst the last teeth to erupt were the mandibular and maxillary first molars. This finding is consistent with the existing literature on the sequence of tooth eruption in dogs [1,2,3]. Humans differ in that the first permanent teeth to emerge are the molars followed by the central incisors and then the lateral incisors [57,58,60,61,62,63,64]. On the maxilla, these are followed by the first premolars and then the canine/second premolars [57,59,60,61,62]. On the mandible, the first premolars/canines are the next teeth to erupt after the incisors, followed by the second premolars [58,60,61,62,63]. The last teeth to emerge are the second molars [57,58,60,61,62,63].

One of the main strengths of this study was the ability to follow the same individuals of a single breed of dog from 8 to 28 weeks of age. In addition, the dogs were exposed to the same environmental factors. However, the study was also subject to limitations. For behavioural reasons, it was sometimes difficult to visualise and capture the dentition statuses of some of the Yorkshire terriers. Furthermore, the dogs were only assessed every four weeks and, therefore, a precise timepoint of loss and eruption could not be determined. Sometimes, it was difficult to distinguish between deciduous and permanent teeth during the stages of mixed dentition. The high incidence of PDT would also have confounded the estimates of age for deciduous tooth loss. As puppies were not acquired until eight weeks of age, and most had their full complement of deciduous teeth, it was not possible to determine the time of eruption for their primary dentition. It was not possible to differentiate the effect of body weight from litter in this study as all the puppies within a litter were of similar sizes and body weights. Additionally, the birth weights of the puppies were not known and their weights at 28 weeks of age may have been influenced by other factors. Finally, this study was relatively small-scale compared to the large data sets available in the human field. Despite this, there are some valuable insights about the impact of dog weight and/or litter on the development of Yorkshire terrier dentition.

Tooth eruption is a complex process and this study has shown that it may be influenced by several factors such as body weight and/or litter. The time of exfoliation of deciduous teeth and eruption of permanent dentition in Yorkshire terriers is variable. The eruption of permanent dentition occurs later in small compared to large Yorkshire terriers, and smaller dogs are also more prone to PDT. This study suggests that the sequence and timing of permanent tooth eruption in Yorkshire terriers may be heritable, and this can have important implications for the risk of PDT, malocclusion, tooth over-crowding, and periodontal disease. For this reason, dogs affected by tooth development disorders should be excluded from future breeding.

## 5. Conclusions

This study has provided information about the age at exfoliation, age at eruption, and prevalence of PDT in Yorkshire terriers, and indicates that litter and body weight can impact these events. It has highlighted the importance of educating owners about potential tooth development problems in Yorkshire terriers and dogs with lower body weight (e.g., less than 3 kg). It is important for Yorkshire terriers to be regularly checked by veterinary professionals between two and seven months of age as their dentition is developing. If PDT are observed, veterinarians and owners must discuss the implications and consider the benefits of surgical extractions. There is need for further research, in a variety of dog breeds, to provide improved guidance to veterinary professionals regarding the “normal” time of eruption and other potential risk factors.

## Figures and Tables

**Table 1:** Variables recorded as part of the study.

Variable	Description
Litter	Dogs’ litter group
Age	Dogs’ age (weeks) at time of measurement
Body weight	Dogs’ weight (kg) at end of study (28 weeks of age)
Tooth type	Incisor, canine, premolar, molar
Persistent deciduous tooth indicator	Defined as whether deciduous teeth were present in the mouth at the same time as their permanent counterparts. Not persistent—0; persistent—1
Surgery indicator	Deciduous dentition, which was not naturally exfoliated by 32 weeks of age and required surgical intervention. No surgery—0; surgery—1

**Table 2:** Average age in weeks (standard deviation) of exfoliation of deciduous dentition by tooth type and body weight.

Tooth Type	Body Weight		
	≤3 kg	>3 kg and ≤5 kg	>5 kg
Incisor	25.8 (2.8)	20.8 (2.5)	20.6 (2.5)
Canine	28.3 (0.6)	25.5 (2.8)	23.8 (2.7)
Premolar	26.9 (2.2)	23.6 (2.7)	23.0 (2.8)

**Table 3:** Average age in weeks (standard deviation) of eruption of permanent dentition by tooth type and body weight.

Tooth Type	Body Weight		
	≤3 kg	>3 kg and ≤5 kg	>5 kg
Incisor	24.4 (2.3)	21.2(2.4)	21.1 (2.5)
Canine	26.8 (1.7)	22.7 (1.6)	22.7 (1.6)
Premolar	26.1 (2.6)	24.1 (3.4)	22.9 (3.0)
Molar	26.4 (2.9)	25.7 (2.9)	24.4 (3.4)

**Table 4:** Proportion of PDT (standard deviation) by tooth type and body weight.

Tooth Type	Body Weight		
	≤3 kg	>3 kg and ≤5 kg	>5 kg
Incisor	0.349 (0.477)	0.017 (0.128)	0.013 (0.115)
Canine	0.891 (0.312)	0.250 (0.433)	0.120 (0.325)
Premolar	0.411 (0.492)	0.061 (0.240)	0.087 (0.281)

**Table 5:** Proportion of PDT (standard deviation), by tooth type and body weight, that required surgical extraction.

Tooth Type	Body Weight		
	≤3 kg	>3 kg and ≤5 kg	>5 kg
Incisor	0.188 (0.390)	0.011 (0.105)	0.003 (0.058)
Canine	0.641 (0.480)	0.167 (0.373)	0.080 (0.271)
Premolar	0.234 (0.424)	0.044 (0.206)	0.080 (0.271)

## Data Availability

Data are not publicly available but can be obtained from the corresponding author upon reasonable request.

## Supplementary Materials

The following supporting information can be downloaded at: https://www.mdpi.com/article/10.3390/vetsci10070406/s1, Table S1: Average age at deciduous tooth loss for each tooth at a body weight of 4.5 kg with lower confidence intervals (LCI) and upper confidence intervals (UCI); Table S2: Estimated age at eruption of permanent dentition at a body weight of 4.5 kg with lower confidence intervals (LCI) and upper confidence intervals (UCI).
