# Supplementary material for: Development of Yorkshire Terrier Dentition
Source: Vet Sci. 2023 Jun 21;10(7):406. doi: 10.3390/vetsci10070406 (PMC10384937; doi:10.3390/vetsci10070406)
Supplement: Supplementary file 1 [file vetsci-10-00406-s001.zip › vetsci-2329067-supplementary.pdf]

Table S1. Average age at deciduous tooth loss for each tooth at a body weight of 4.5 kg with lower (LCI) and upper confidence interval limits (UCI). Tooth numbers represent maxillary first, second and third incisors (501-503, 601-603), canine (504, 604) and premolars (506-508, 606-608) and mandibular first, second and third incisors (701-703, 801-803), canine (704, 804) and premolars (706-708, 806-808).

Tooth Number	Average Age (weeks)	LCI	UCI
802	20.59	19.36	21.90
702	20.59	19.37	21.89
701	20.76	19.51	22.08
801	20.83	19.57	22.17
703	20.92	19.69	22.23
601	21.39	20.06	22.81
602	21.45	20.15	22.83
803	21.47	20.21	22.81
501	21.60	20.25	23.04
502	21.78	20.45	23.20
607	22.74	21.38	24.19
608	22.91	21.53	24.39
707	23.08	21.69	24.55
507	23.12	21.71	24.61
807	23.20	21.80	24.70
508	23.26	21.82	24.78
708	23.84	22.33	25.45
808	24.04	22.50	25.68
603	24.42	22.82	26.14
503	24.60	23.03	26.29
806	24.78	23.22	26.45
804	25.18	23.47	27.02
704	25.28	23.54	27.15
606	25.43	23.81	27.16
706	25.45	23.81	27.20
506	25.88	24.19	27.70
504	27.68	25.55	29.99
604	27.70	25.49	30.10

Table S2. Estimated average age at eruption of permanent dentition at a body weight of 4.5 kg with lower (LCI) and upper confidence limits (UCI). Tooth numbers represent maxillary first, second and third incisors (101-103, 201-203), canine (104, 204), premolars (105-108, 205-208) and molars (109-110, 209-210) and mandibular first, second and third incisors (301-303, 401-403), canine (304, 404), premolars (305-308, 405-408) and molars (309-311, 409-411).

Tooth Number	Average Age (weeks)	LCI	UCI
201	21.20	19.67	22.85
301	21.39	19.78	23.12
401	21.43	19.87	23.11
101	21.68	20.12	23.35
302	21.71	20.08	23.47
202	21.72	20.16	23.41
402	21.74	20.13	23.48
403	22.69	21.07	24.43
102	22.72	21.13	24.43
108	22.81	21.24	24.49
208	22.92	21.33	24.63
309	23.17	21.35	25.14
303	23.25	21.61	25.00
204	23.30	21.64	25.10
104	23.32	21.66	25.11
107	23.42	21.80	25.15
304	23.47	21.76	25.31
207	23.76	22.05	25.60
409	23.85	22.12	25.71
308	23.92	22.23	25.73
408	23.94	22.26	25.75
404	23.98	22.27	25.83
109	24.03	22.08	26.16
407	24.05	22.40	25.82
307	24.16	22.50	25.94
105	24.23	22.28	26.35
103	24.37	22.58	26.31
203	24.40	22.62	26.31
209	24.65	22.56	26.92
205	24.68	22.69	26.84
206	25.37	23.42	27.49
406	25.50	23.59	27.57
306	25.57	23.74	27.54
310	25.70	23.62	27.96
410	25.88	23.76	28.19
110	25.98	23.67	28.53
106	26.01	23.85	28.35
210	27.09	24.73	29.67
305	27.14	24.88	29.62
405	27.29	25.06	29.71
411	33.40	27.82	40.09
311	35.67	27.10	46.95
